# Elementary school physical activity opportunities and physical fitness of students: A statewide cross-sectional study of schools

**DOI:** 10.1371/journal.pone.0210444

**Published:** 2019-01-15

**Authors:** Patricia C. Cheung, Padra A. Franks, Michael R. Kramer, Christi M. Kay, Carolyn D. Drews-Botsch, Jean A. Welsh, Julie A. Gazmararian

**Affiliations:** 1 Department of Epidemiology, Emory University, Atlanta, Georgia, United States of America; 2 HealthMPowers, Norcross, Georgia, United States of America; 3 School of Medicine, Emory University, Atlanta, Georgia, United States of America; TNO, NETHERLANDS

## Abstract

**Background:**

Using a cross-sectional design, we assessed the relationship between the time schools provide for physical activity and the proportion of students achieving a healthy aerobic capacity or body mass index.

**Methods:**

In 2013–2014, physical education and grade-level teachers from 905 of 1,244 Georgia elementary schools provided survey data about the frequency and duration of physical activity opportunities offered before, during, and after school. Log-binomial models related the weekly physical activity minutes provided by schools to the proportion of children in the FitnessGram healthy fitness zone for aerobic capacity or body mass index while adjusting for school characteristics and demographics.

**Results:**

During-school physical activity time was not associated with student fitness, but schools with before-school physical activity programs had a moderately higher prevalence of healthy aerobic capacity (prevalence ratio among girls: 1.06; 99% confidence interval: 1.00–1.13; prevalence ratio among boys: 1.03; 99% confidence interval: 0.99–1.08). Each additional 30 minutes of recess per week was associated with no more than a 3%-higher proportion of students with healthy body mass indexes (prevalence ratio among girls: 1.01; 99% confidence interval: 1.00–1.03; prevalence ratio among boys: 1.01; 99% confidence interval: 0.99–1.03).

**Conclusions:**

The amount of physical activity time provided by schools is not strongly associated with school-aggregated student fitness. Future studies should be designed to assess the importance of school-based physical activity time on student fitness, relative to physical activity type and quality.

## Introduction

Poor health-related fitness in childhood increases future heart disease risk by lowering high-density lipoproteins and increasing triglycerides, elevating blood pressure, and causing arterial stiffness and early mortality [1–5]. However, results from FitnessGram, a widely used school-based, health-related fitness assessment, show that only 61–62% of elementary-aged children in the US meet healthy fitness zone (HFZ) standards for aerobic capacity and 53–60% of students meet the HFZ for body mass index (BMI) [6]. Schools serve 98% of US children between 7–13 years [7], and during the week, children are at schools for half of their waking hours [8]. As a result, the Institute of Medicine recommends that more than half of the recommended 60 minutes of moderate-to-vigorous physical activity (MVPA) per day should be performed during the regular school day [9].

Almost all US state policies mandate elementary school physical activity (PA) by specifying the amount of time schools offer for PA [10]. However, few researchers have examined the relationship between school-based PA opportunity time and student fitness, particularly in the elementary school setting [10]. Researchers using an ecologic design among low-income California middle schools found that schools providing more PA opportunities on school grounds outside of school time (e.g. after-school PA) had children with higher aerobic capacity than schools providing fewer such opportunities [11]. In the Early Child Longitudinal Study-Kindergarten cohort, additional recess time, but not physical education (PE) time, was associated with a decrease in BMI between kindergarten and the fifth grade [12]. In contrast, other researchers have reported that offering more PE to fifth-grade students was associated with lower BMI z-scores [13], and still others have concluded that PA resources and programs are not associated with fifth-grade student BMI [14]. Due to the sparse literature and mixed findings about the relationship of PA opportunity time with child fitness, the objective of this cross-sectional study was to assess the association between school-level PA opportunities (PE time, recess time, in-class PA time, before-school PA, and after-school PA) and school-aggregated fitness levels of Georgia elementary students. It was hypothesized that schools offering more PA opportunities would have higher proportions of students with healthy fitness levels.

## Materials and methods

### Procedure

One administrator, one PE teacher, and one grade-level chair from each grade of all 1,244 Georgia public elementary schools educating fifth-grade students or younger were sent a survey between October 2013 and September 2014 with the goal of estimating the amount of time PA was offered before, during (PE, recess, in-class PA breaks), and after school each week. The online survey was created by a multidisciplinary team and adapted from other widely-used and reliability-tested school PA survey tools [9,15–20]. PE teachers provided data about PE, and before-school and after-school PA. Grade-level chairs provided data about recess and in-class PA breaks. Administrator data were not used in the current study since survey items assigned to administrators were not relevant for the current research questions. Data were analyzed in 2016–2017. The study was reviewed by the Emory University Institutional Review Board and determined to be exempt.

### Instrumentation

Publicly available 2013–2014 Georgia FitnessGram records provided fitness data [21,22]. In Georgia elementary schools, the annual test is composed of five assessments, but only Progressive Aerobic Cardiovascular Endurance Run (PACER) and BMI, measures of aerobic capacity and body composition (measures most strongly linked to health outcomes [23–27]), were included in the current study. In Georgia, all assessments are required for fourth- and fifth-grade students, but only body composition is required for first- to third-grade students. Publicly available data include the number of youth classified in the HFZ for each test event and the number of students taking each test event at each school, stratified by sex. Both aerobic capacity and BMI HFZ cutoffs were derived from studies using National Health and Nutrition Examination Survey data between 1999 and 2004 to assess the risk of metabolic syndrome in youth [28,29].

Exposure variables were obtained from School PA Survey questions assessing PA opportunities at each grade level [20]. These questions evaluated the frequency of PE and recess (0, 1, 2, 3, 4, or 5 days per week). Separate questions assessed the duration of each PE (<15, 15–19, 20–29, 30–39, 40–49, or ≥50 minutes per PE class) and recess period (<15, 15–19, 20–29, or ≥30 minutes per recess). We also evaluated in-class PA time (0, 1–5, 6–10, 11–15, 16–20, 21–25, or >25 minutes per day) and presence or absence of PA opportunities before (yes/no) and after school (yes/no; the survey did not assess the duration of before- and after-school PA opportunities). To approximate continuous estimates of PA time (minutes/week) from categorical responses for each during-school PA opportunity (PE, recess, and in-class PA breaks), a Monte Carlo simulation was used (S1–S3 Appendices) [30]. Since aerobic capacity was only assessed among fourth- and fifth-grade students, but body composition was assessed among first- through fifth-grade students, non-missing grade-specific PA opportunity times were averaged between the fourth and fifth grades (182,361 students when assessing aerobic capacity) or averaged across the first to fifth grades (462,556 students when assessing body composition). During-school PA time was defined as the sum of the estimated time spent in PE, recess, and in-class PA breaks across each week.

Covariates included school-level demographics (free/reduced lunch rate [FRL] and race/ethnicity) and other school-level characteristics (school size and geographical setting) [22,31]. Prior National Center for Education Statistics publications informed nominal FRL (≤25%, 25.1–50%, 50.1–75%, >75%) and geographical setting (city, suburb, town, rural) categories [31]. When no a priori categorizations were identified, covariates were retained as continuous variables (e.g. percent White, percent Hispanic, and school size). Race/ethnicity could be characterized almost completely by percent White, Black, and Hispanic (the sum of percent White, Black, and Hispanic was almost always 100%), so percent Black was excluded to serve as the referent group in adjusted models as it did not provide much additional information in the adjusted models beyond percent White and Hispanic.

In the current study, six unique respondents were requested to complete the survey within each school (first- through fifth-grade-level chairs and a PE teacher). Some schools did not have all six respondents which could have biased results. To address these missing data, prior to Monte Carlo simulation in the main analyses described above, SAS PROC MI with fully conditional specification was used to impute five values for each missing observation of frequency and duration of PE, frequency and duration of recess, in-class PA, before-school PA, after-school PA, FRL, percent White, percent Hispanic, school size, and geographical setting.

### Data analysis

First, descriptive statistics (mean and standard deviations, or sample sizes and frequencies) were calculated for demographics and other school covariates, exposures (PA opportunities), and outcomes (aerobic capacity and body composition HFZ prevalence). Second, bivariate analyses were conducted to examine the aerobic capacity and body composition HFZ prevalence by demographics and other school characteristics. In the bivariate analyses, HFZ prevalence estimates were reported at the 33^rd^ and 66^th^ percentiles for percent White, percent Hispanic, and school size since these covariates were continuous variables.

In the main analyses, unadjusted and adjusted models controlling for demographics and other school characteristics were computed to assess the relationship between school PA opportunities and school-level HFZ prevalence. Log-binomial models (or log-Poisson models when log-binomial models did not converge) estimated prevalence ratios (PRs). For continuous exposures (during-school PA, PE, recess, and in-class PA break time), PRs can be interpreted as the relative change in HFZ prevalence associated with each 30-minute increase in PA opportunity time. For before- and after-school exposures, PRs can be interpreted as the HFZ prevalence among schools with before/after school PA programs compared with the HFZ prevalence among schools without a before/after school PA program. For analyses involving PA opportunity times, estimates from each Monte Carlo simulation were summarized using PROC MIANALYZE to obtain PRs and 99% CIs.

For all models, schools were weighted by the number of students in the school because some schools conducted and reported FitnessGram data on more than one occasion within the same school year, overestimating the number of students taking the spring semester test. Additive and multiplicative interaction between PA time and all covariates were tested, and significant additive and multiplicative interaction was noted across school size and geographical setting, but stratum-specific estimates and CIs largely overlapped [32], so overall estimates are presented. All analyses were stratified by gender, as prior literature has suggested that fitness and PA differ by gender [6,33]. To account for multiple testing and to be consistent with prior studies, alpha was set at 0.01 [20]. Analyses were also repeated using alpha at 0.05, and significance was consistent across both alphas. All analyses were performed using SAS version 9.4 (Cary, NC).

## Results

Of the 1,244 eligible Georgia elementary schools, 68 schools were excluded because HFZ prevalence data were missing and 271 schools were excluded because the School PA Survey was not completed by either a PE teacher or any grade-level chairs. Analyzed schools (905 schools; 73%) were more likely to have more than 25% FRL, have a larger proportion of white and Hispanic (but fewer black) students, have a larger student body, and be located in suburban areas than excluded schools [32].

Forty-five percent of analyzed schools reported that more than 75% of children received FRL; only 11% of schools reported that one-quarter or fewer of their students received FRL (Table 1). The average school had 42% white, 37% black, and 14% Hispanic students. On average, schools had 648 students, and the largest proportion of schools was located in a suburban area (42%). Most PA time offered during the school day came from PE (mean: 100 minutes/week). Across the state, the average proportion of girls and boys meeting aerobic capacity HFZ at each school was 61% and 71% respectively. The average school prevalence of healthy body composition was 59% for both boys and girls.

**Table 1:** Demographic and school characteristics of participating Georgia elementary schools in 2013–2014 (n = 905 schools).

Characteristic			N or Mean	% or SD
**Free/reduced lunch rate (n, %)**				
	Low (≤25% FRL)		97	10.7
	Mid-low (25%<FRL≤50%)		144	15.9
	Mid-high (50%<FRL≤75%)		253	28.0
	High (>75% FRL)		411	45.4
**Mean (SD) % White**			42.0	29.1
**Mean (SD) % Black**			37.2	30.1
**Mean (SD) % Hispanic**			13.7	15.8
**Mean (SD) school size (students)**			647.7	230.2
**School geographical location (n, %)**				
	City		178	19.7
	Suburban		382	42.2
	Town		94	10.4
	Rural		251	27.7
**Mean (SD) during-school physical activity opportunity time** ^**a**^			216.2	58.7
	Physical education time (minutes/week)		99.6	46.2
	Recess time (minutes/week)		86.4	34.6
	In-class physical activity (minutes/week)		29.9	18.4
	% of schools offering before-school physical activity		118.0	13.0
	% of schools offering after-school physical activity		300.0	33.2
**Mean (SD) % of students at each school in HFZ**				
	Aerobic capacity among females		61.2	23.9
	Aerobic capacity among males		71.2	19.9
	Body composition among females		59.3	10.3
	Body composition among males		59.3	10.0

FRL = Free/reduced lunch rate; SD = Standard deviation; HFZ = Healthy fitness zone

^a^Generated from the Monte Carlo simulation. During-school PA time may not equal the exact sum of PE time, recess time, in-class PA time, before-school PA time, and after-school PA time because of randomness generated from the Monte Carlo model and missing variables.

Schools with 25% or fewer students receiving FRL had the highest proportion of students with healthy aerobic capacity (77–85%) and body composition (71–74%), while schools with more than three-quarters of students receiving FRL had the lowest proportions (58–68% for aerobic capacity and 55% for body composition; Table 2). Schools with a large proportion (60%) of white students had a higher proportion of students with a healthy aerobic capacity (67–76%) and healthy body composition (63–64%) than schools with a lower proportion (23%) of white students (63–72% of students with healthy aerobic capacity and 57–58% with healthy body composition). Schools in suburban areas had the highest prevalence of healthy aerobic capacity (68–77%) and body composition (61–63%) while schools in towns had the lowest proportion of students with healthy aerobic capacity (57–67%) and body composition (57–58%).

**Table 2:** Mean school prevalence of healthy fitness across Georgia elementary school characteristics in 2013–2014 (n = 905 schools).

Characteristic		Aerobic capacity						Body mass index					
		Girls			Boys			Girls			Boys		
		Mean %	99% CI		Mean %	99% CI		Mean %	99% CI		Mean %	99% CI	
**Free/reduced lunch rate**													
	Low (≤25%)	76.9	(71.1	83.3)	84.7	(80.5	89.2)	73.5	(70.5	76.6)	70.8	(68.0	73.8)
	Mid-low (25%<FRL≤50%)	70.0	(64.6	75.9)	78.3	(74.1	82.8)	65.1	(63.2	67.0)	64.1	(62.5	65.8)
	Mid-high (50%<FRL≤75%)	64.3	(60.7	68.1)	73.8	(71.1	76.6)	59.3	(57.8	60.7)	58.6	(57.2	60.0)
	High (>75%)	58.2	(53.4	63.5)	68.4	(64.4	72.6)	54.9	(53.5	56.2)	54.7	(53.0	56.4)
**Mean % White**^**a**^													
	Low % White	62.6	(59.0	66.4)	72.2	(69.3	75.2)	58.0	(56.9	59.2)	57.1	(55.8	58.4)
	High % White	67.2	(64.4	70.0)	75.9	(73.7	78.2)	63.8	(62.6	65.1)	62.9	(61.8	64.0)
**Mean % Hispanic**^**b**^													
	Low % Hispanic	65.4	(62.5	68.4)	74.6	(72.2	77.1)	62.9	(61.5	64.3)	63.0	(61.8	64.2)
	High % Hispanic	65.3	(62.7	68.0)	74.5	(72.4	76.6)	61.7	(60.6	62.9)	61.2	(60.2	62.2)
**School size**^**c**^													
	Small school	63.6	(60.6	66.8)	72.9	(70.5	75.4)	60.1	(58.3	61.9)	60.5	(58.6	62.3)
	Large school	64.7	(62.2	67.2)	73.9	(71.9	76.0)	60.6	(59.6	61.7)	60.2	(59.2	61.2)
**School geographical location**													
	City	66.4	(61.2	72.0)	74.9	(71.0	79.0)	59.9	(56.8	63.1)	59.6	(56.7	62.7)
	Suburban	67.5	(63.7	71.5)	76.6	(73.8	79.6)	62.5	(60.8	64.4)	60.6	(58.7	62.5)
	Town	57.2	(49.4	66.2)	67.3	(60.1	75.5)	56.8	(54.0	59.8)	58.3	(55.8	61.0)
	Rural	63.6	(59.4	68.1)	72.7	(69.3	76.3)	59.4	(57.8	61.1)	59.7	(58.1	61.4)

FRL = Free/reduced lunch rate; CI = Confidence interval.

^a^Prevalence reported at the 33^rd^ and 66^th^ percentiles (23%, 60% White).

^b^Prevalence reported at the 33^rd^ and 66^th^ percentiles (5%, 12% Hispanic).

^c^Prevalence reported at the 33^rd^ and 66^th^ percentiles (524, 700 students).

### Aerobic capacity

Across all analyses, neither during-school PA time, nor the amount of time provided for PE, recess, or in-class PA breaks were associated with aerobic capacity (Table 3). In unadjusted models, schools offering before-school PA programs had an 8–12% higher prevalence of children in the HFZ for aerobic capacity. For example, the estimated prevalence of healthy aerobic capacity was 71% among girls and 79% among boys in schools with before-school PA programs, compared with 64% and 73% among girls and boys in schools without before-school PA programs. Although schools with before-school PA programs tended to have a higher prevalence of children in the aerobic capacity HFZ, after adjustment for school-level characteristics, this relationship was attenuated (aPR_girls_: 1.06; 99% CI: 1.00–1.13; aPR_boys_: 1.03; 99% CI: 0.99–1.08). The adjusted prevalence of healthy aerobic capacity was also moderately higher among schools with after-school PA than schools without after-school PA among girls (aPR_girls_: 1.04; 99% CI: 0.98–1.11; aPR_boys_: 1.03; 99% CI: 1.00–1.07), but this was not statistically significant.

**Table 3:** Prevalence ratios (PRs) comparing the prevalence of students with healthy aerobic capacity across school physical activity.

		Girls						Boys					
		PR	99% CI		aPR^a^	99% CI		PR	99% CI		aPR^a^	99% CI	
**During-school physical activity**^**b**^^,^ ^**c**^		1.01	(0.99	1.03)	1.00	(0.98	1.01)	1.00	(0.99	1.02)	1.00	(0.98	1.02)
	Physical education^b^	0.99	(0.97	1.02)	1.00	(0.97	1.02)	1.00	(0.98	1.01)	1.00	(0.98	1.02)
	Recess^b^	1.02	(0.99	1.06)	1.00	(0.96	1.04)	1.02	(0.99	1.04)	1.00	(0.97	1.02)
	In-class physical activity breaks^b^	1.00	(0.94	1.06)	0.99	(0.94	1.05)	1.00	(0.96	1.04)	1.00	(0.96	1.03)
**Before-school PA program**^**d**^		**1.12**	**(1.03**	**1.21)**	1.06	(1.00	1.13)	**1.08**	**(1.02**	**1.14)**	1.03	(0.99	1.08)
**After-school PA program**^**d**^		1.04	(0.96	1.13)	1.04	(0.98	1.11)	1.02	(0.97	1.08)	1.03	(1.00	1.07)

opportunities (n = 905; 2013–2014).

CI = Confidence interval; PA = Physical activity

^a^Adjusted for free/reduced lunch rate, percent White, percent Hispanic, school size, geographical setting (city, suburban, town, rural)

^b^PR indicates the change in healthy fitness zone prevalence associated with each additional 30 minutes of physical activity per week

^c^During-school PA time may not equal the exact sum of PE time, recess time, and in-class PA time because of randomness generated from the Monte Carlo model or missing variables

^d^PR indicates the additional healthy fitness zone prevalence associated with presence (versus absence) of a program

**Boldface** indicates significance (p<0.01)

### Body composition

During-school PA, PE, in-class PA, and before- and after-school PA were not associated with the prevalence of children in the HFZ for body composition (Table 4). Crude analyses showed that schools with the 25^th^ percentile recess time offered 67 minutes of recess per week and reported 59% of girls and boys to have healthy body composition, while schools with the 75^th^ percentile recess time (108 minutes per week) had 62% of girls and 61% of boys in the HFZ for body composition. After accounting for demographics and other school characteristics, the association between recess and healthy body composition was attenuated: After adjustment, schools offering 67 minutes of recess per week reported 58% of girls and 60% of boys to have healthy body composition, while schools providing 108 minutes of recess per week had 59% of girls and 61% of boys in HFZ for body composition (aPR_girls_: 1.01; 99% CI: 1.00–1.03; aPR_boys_: 1.01; 99% CI: 0.99–1.03).

**Table 4:** Prevalence ratios (PRs) comparing the prevalence of students with healthy body composition across school physical activity opportunities (n = 905; 2013–2014).

		Girls						Boys						
		PR	99% CI		aPR^a^	99% CI		PR	99% CI		aPR^a^	99% CI		
**During-school physical activity**^**b**^^,^ ^**c**^		1.02	(1.00	1.03)	1.00	(0.99	1.01)	1.02	(1.00	1.03)	1.01	(0.99		1.02)
	Physical education^b^	1.00	(0.99	1.02)	1.00	(0.99	1.01)	1.01	(1.00	1.03)	1.00	(0.99		1.02)
	Recess^b^	**1.04**	**(1.02**	**1.05)**	1.01	(1.00	1.03)	**1.03**	**(1.01**	**1.04)**	1.01	(0.99		1.03)
	In-class physical activity breaks^b^	0.99	(0.96	1.03)	1.00	(0.97	1.02)	0.99	(0.95	1.03)	1.00	(0.97		1.02)
**Before-school PA program**^**d**^		1.05	(0.99	1.12)	1.02	(0.99	1.05)	1.03	(0.95	1.11)	1.02	(0.99	1.05)	
**After-school PA program**^**d**^		0.97	(0.94	1.01)	0.99	(0.97	1.01)	0.97	(0.93	1.02)	1.00	(0.97	1.02)	

CI = Confidence interval; PA = Physical activity.

^a^Adjusted for free/reduced lunch rate, percent White, percent Hispanic, school size, geographical setting (city, suburban, town, rural).

^b^PR indicates the change in healthy fitness zone prevalence associated with each additional 30 minutes of physical activity per week.

^c^During-school PA time may not equal the exact sum of PE time, recess time, and in-class PA time because of randomness generated from the Monte Carlo model or missing variables.

^d^PR indicates the additional healthy fitness zone prevalence associated with presence (versus absence) of a program.

**Boldface** indicates significance (p<0.01).

## Discussion

The amount of time schools provide in recess, PE and in-class PA, and the presence of before- and after-school PA opportunities may have a positive but minimal effect on the proportion of children with healthy levels of aerobic capacity and body composition at each school. Offering before-school PA programs may be associated with an increased prevalence of children with healthy aerobic capacity, but it does not seem to be associated with an increased prevalence of children with BMIs in the HFZ. In contrast, increased recess time may be associated with a higher prevalence of children with healthy BMIs at each school.

Findings from the current study contrast with prior US literature reporting an association between PE or before/after-school PA time and aerobic capacity in middle and elementary schools [11,34,35]. One potential explanation is that prior researchers used other measures of aerobic capacity such as mile-run time, while we used PACER outcomes conceptualized as binary HFZ categories in the current study. Beyond the decreased power when relying on categorized variables, classification agreement between PACER and one-mile run/walk tests also varies by child characteristics and environmental conditions [36]. Only a few studies on the relationship between elementary school PA opportunities and student body composition have been published, and similar to the current study, almost all have reported modest or null findings [12–14].

School-based PA opportunity time may not influence student aerobic capacity and BMI as much as PA and dietary behaviors outside of the educational system. Recently, researchers found that school-level median household income, but not PA resources/programs, was inversely associated with BMI among girls [14]. Many obesogenic risk factors inside and outside of the school PA environment have been linked with low socioeconomic status, including insufficient PA, poor nutrition as well as high consumption of sugar-sweetened beverages and fruit juices [37,38]. Researchers have noted that differences between schools only account for a small part of the total variation in aerobic capacity (intraclass correlation coefficient: 0.10) and student BMI percentile or percent of overweight/obese students (intraclass correlation coefficients: 0.03–0.10), because energy intake and expenditure also occur outside of school [12,39,40]. Further, it has long been suggested that exercise interventions may lead to compensatory responses in the form of increased energy intake or decreased energy expenditure through exercise and non-exercise activity outside of school [41,42].

Another possible explanation for the moderate and null findings may be that the quality of PA offered may vary between schools providing the same frequency and duration of PA [43]. Researchers assessing PA programs designed to increase PA quality have consistently reported improved student aerobic capacity [44], and a larger proportion of PA time spent in MVPA is associated with greater energy expenditure [45]. However, researchers using accelerometer-measured MVPA have reported that students spend only 33% of time during PE class performing MVPA, and it is possible that time spent in MVPA is just a fraction of the PA opportunity time reported in this study [46]. Further, characteristics of the child and school environment (e.g. professional development for PE teachers) are associated with PA quality [47,48].

### Strengths

This study had at least three strengths. First, this study included a large number of schools, with relatively high response rates on the School PA Survey (73%) and FitnessGram (95%) compared to similar studies conducted in school settings. Second, this statewide study involved diverse settings, increasing the generalizability of our findings. Finally, we built on existing knowledge by identifying the effects of specific PA opportunities offered to students during the elementary school day on physical fitness.

### Limitations

Despite the strengths of this study, there were at least four limitations. First, the cross-sectional design did not exclude the possibility of reverse causation, where student fitness may have influenced PA opportunities provided by schools. In addition, it is possible that elementary school PA opportunities influence fitness in the long term [12], which could not be assessed using a cross-sectional design. Second, the School PA Survey has not yet been validated, and differences between scheduled and offered PA time may have occurred because of teacher availability and special events [45]. While cost and limited resources precluded direct observation of school-based PA time in this statewide study, PA policy assessments on which the School PA Survey was based have demonstrated *moderate* to *almost perfect* intra-rater reliability [19,49]. Further, inter-rater reliability for the School PA Survey itself has been estimated to be *fair* to *almost perfect* [20]. The survey was adopted from widely used surveys by a large multidisciplinary team and our group is currently exploring a formal study assessing survey validity and reliability. Third, to maximize school response, the School PA Survey was open for 10 months, and the timing of early versus late responses could have affected estimates. However, with the exception of the presence of after-school PA opportunities, none of the reported PA opportunities differed by season of survey completion [32]. Finally, fitness data were aggregated at the school level, and aside from sex, we were unable to assess for differences across other individual demographic, behavioral, and health characteristics.

## Conclusions

In this study, we demonstrated the variation in the proportion of students with healthy aerobic capacity and body composition across school socioeconomic status, geographical location, and racial composition, supporting the need to address health disparities across school demographics and settings. At the individual level, time spent engaged in PA has been shown to improve fitness [50,51]. In contrast, we suggest that the presence of PA programs and the amount of time offered by schools for PA are only moderately associated with the proportion of healthy student fitness at the school level. Increased quality of PA, as measured by percent time spent in MVPA or type of PA performed within the time allocated for PA, is likely required for achieving higher proportions of students with healthy fitness levels [43]. If other researchers find that improved quality of PA, in addition to the amount of time PA is offered by elementary schools, is important for improving student fitness, then current school PA policies, which are mostly based on time, and their implications for student health should be re-examined.

## Supporting information

S1 Appendix Supplemental methods. (DOCX)

S2 Appendix Joint distribution of physical education frequency and duration for Georgia fifth-grade students (2013–2014). (DOCX)

S3 Appendix Histogram of physical education (PE) time (minutes per week) across Georgia elementary schools to demonstrate the creation of the continuous PE time variable. (DOCX)
