# Supplementary material for: Elementary school physical activity opportunities and physical fitness of students: A statewide cross-sectional study of schools
Source: PLoS One. 2019 Jan 15;14(1):e0210444. doi: 10.1371/journal.pone.0210444 (PMC6333378; doi:10.1371/journal.pone.0210444)
Supplement: S1 Appendix — (DOCX) [file pone.0210444.s001.docx]

**S1 Appendix**

**Supplemental Methods**

To approximate continuous estimates of PA time (minutes/week) from categorical responses for each during-school PA opportunity (PE, recess, and in-class PA breaks), a Monte Carlo simulation was used [30]. For PE and recess, each replicate used a Monte Carlo sampling approach assuming a uniform distribution within each category to randomly assign a duration (minutes/occurrence), which was multiplied by its frequency (occurrences/week) to obtain minutes of PA per week (Appendices S2-S3) [30]. For in-class PA time, each replicate randomly drew an in-class PA time within each category. This was repeated to produce 500 estimates. While an alternative would have been to use the midpoint of PA frequency and duration intervals to calculate the weekly PA opportunity time, the Monte Carlo simulation was reported since confidence intervals (CIs) were more likely to correspond to the uncertainty in PA opportunity estimation from categorical survey responses.
