# Supplementary material for: Elementary school physical activity opportunities and physical fitness of students: A statewide cross-sectional study of schools
Source: PLoS One. 2019 Jan 15;14(1):e0210444. doi: 10.1371/journal.pone.0210444 (PMC6333378; doi:10.1371/journal.pone.0210444)
Supplement: S2 Appendix — (DOCX) [file pone.0210444.s002.docx]

**S2 Appendix**

| **S2 Appendix.** Joint distribution of physical education (PE) frequency and duration for Georgia fifth-grade students (2013-2014). | | | | |
| --- | --- | --- | --- | --- |
| **PE frequency (classes per week)** | **PE duration (minutes per class)** | | | |
|  | **20-29 minutes**  **(n [freq])** | **30-39 minutes**  **(n [freq])** | **40-49 minutes (n [freq])** | **> 50 minutes**  **(n [freq])** |
| **1** | 0 (0.00%) | 6 (0.80%) | 144 (19.3%) | 21 (2.82%) |
| **2** | 2 (0.27%) | 1 (1.34%) | **291 (39.01%)^a^** | 71 (9.52%) |
| **3** | 1 (0.13%) | 5 (0.67%) | 104 (13.94%) | 28 (3.75%) |
| **4** | 2 (0.27%) | 4 (0.54%) | 12 (1.61%) | 5 (0.67%) |
| **5** | 0 (0.00%) | 12 (1.61%) | 19 (2.55%) | 8 (1.07%) |
| PE=physical education  ^a^Schools offering two days of PE for 40-49 minutes per class is **boldfaced** to demonstrate the creation of the continuous PE time variable. Schools in this group provided an average of 89.60±5.05 (range=80.04-97.96) minutes of PE per week. | | | | |
