# Supplementary material for: Elementary school physical activity opportunities and physical fitness of students: A statewide cross-sectional study of schools
Source: PLoS One. 2019 Jan 15;14(1):e0210444. doi: 10.1371/journal.pone.0210444 (PMC6333378; doi:10.1371/journal.pone.0210444)
Supplement: S3 Appendix — (DOCX) [file pone.0210444.s003.docx]

**S3 Appendix**

*
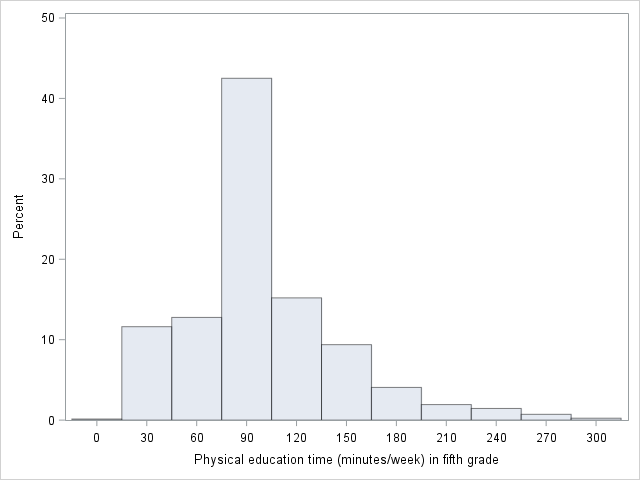
*

**S3 Appendix.** Histogram of physical education (PE) time (minutes per week) across Georgia elementary schools to demonstrate the creation of the continuous PE time variable.
